# Oxidosqualene cyclases involved in the biosynthesis of triterpenoids in *Quercus suber* cork

**DOI:** 10.1038/s41598-020-64913-5

**Published:** 2020-05-15

**Authors:** Lucas Busta, Olga Serra, Ok Tae Kim, Marisa Molinas, Irene Peré-Fossoul, Mercè Figueras, Reinhard Jetter

**Affiliations:** 1 Department of Chemistry, University of British Columbia, 2036 Main Mall, Vancouver, BC V6T 1Z4 Canada; 2 Center for Plant Science Innovation and Department of Biochemistry, University of Nebraska–Lincoln, Lincoln, Nebraska 68588 USA; 3 Laboratori del Suro, Department of Biology, Facultat de Ciències, Universitat de Girona, Campus Montilivi sn. 17071, Girona, Spain; 4 Department of Herbal Crop Research, National Institute of Horticultural and Herbal Science, RDA, Eumseong, 369-873 South Korea; 5 Department of Botany, University of British Columbia, 6270 University Boulevard, Vancouver, BC V6T 1Z4 Canada

**Keywords:** Biosynthesis, Enzymes, Lipids, Natural products

## Abstract

Cork is a water-impermeable, suberin-based material harboring lignin, (hemi)cellulose, and extractable small molecules (primarily triterpenoids). Extractables strongly influence the properties of suberin-based materials. Though these previous findings suggest a key role for triterpenoids in cork material quality, directly testing this idea is hindered in part because it is not known which genes control cork triterpenoid biosynthesis. Here, we used gas chromatography and mass spectrometry to determine that the majority (>85%) of non-polar extractables from cork were pentacyclic triterpenoids, primarily betulinic acid, friedelin, and hydroxy-friedelin. In other plants, triterpenoids are generated by oxidosqualene cyclases (OSCs). Accordingly, we mined *Quercus suber* EST libraries for OSC fragments to use in a RACE PCR-based approach and cloned three full-length OSC transcripts from cork (*QsOSC1*-*3*). Heterologous expression in *Saccharomyces cerevisiae* revealed that *QsOSC1*-*3* respectively encoded enzymes with lupeol synthase, mixed α- and β-amyrin synthase, and mixed β-amyrin and friedelin synthase activities. These activities together account for the backbone structures of the major cork triterpenoids. Finally, we analyzed the sequences of QsOSC1-3 and other plant OSCs to identify residues associated with specific OSC activities, then combined this with analyses of *Q. suber* transcriptomic and genomic data to evaluate potential redundancies in cork triterpenoid biosynthesis.

## Introduction

Cork is a naturally occurring, renewable, sustainable biological material found in the outer bark of diverse tree species. Commercial cork is harvested from the cork oak (*Quercus suber* L.) via the periodic removal of its outer bark, which is also called phellem. Once dried and processed, the bark yields a material that is flame-resistant, buoyant, elastic, and impermeable to water^1^. These remarkable properties have led to the widespread use of cork in the creation of, for example, building materials, floats, and bottle stoppers. The diverse industrial uses of cork highlight the importance of understanding the biochemical and genetic basis for the material’s physical properties.

As with other naturally occurring biological materials, the chemical composition of cork has a major influence on its physical properties^1^. Previous studies have revealed that cork comprises four main classes of chemicals: suberin, lignin, (hemi)cellulose, and small-molecule extractables^2^. While the relative proportions of these components can vary between cork isolates^3,4^, suberin is, on average, the most abundant component (~40%), with the other three contributing roughly equally (~20% each) to the total. Of the four chemical classes constituting cork tissue, suberin, lignin, and (hemi)cellulose are all polymers found in essentially all vascular plant lineages. In contrast, the extractables comprise primarily triterpenoids and phenolics - metabolites that accumulate in pronounced lineage-specific patterns^5^. Unlike most enzymes involved in ubiquitous metabolic processes (“primary” metabolism), lineage-specific metabolism (a.k.a., “specialized” or “secondary” metabolism) is often mediated by relatively promiscuous enzymes^6^. In these enzymes, single amino acid substitutions can have profound effects on substrate and product profiles^7–9^. Thus, accurately predicting the precise catalytic activities of specialized metabolic enzymes is notoriously difficult, and targeted analyses of such enzymes are required in order to determine product profiles. This means that careful functional characterization of genes controlling extractables biosynthesis is necessary to understand the biochemical as well as genetic basis for cork formation, and eventually its material properties.

Previous analyses of phellem (cork) extractables revealed that they comprise primarily non-polar compounds (in *Q. suber* cork, mainly triterpenoids along with smaller amounts of fatty acyl-derived compounds; sometimes called suberin-associated waxes)^10^, together with smaller amounts of polar constituents (in *Q. suber* cork, phenolics)^11–15^. Triterpenoids are a group of diverse natural products originating from six acetyl-CoA-derived isopentenyl diphosphate units supplied by the cytosolic mevalonate pathway^16^. The first diversifying step in triterpenoid biosynthesis is the cyclization of 2,3-oxidosqualene, catalyzed by an oxidosqualene cyclase (OSC). OSCs have diversified considerably across vascular plants, and more than 100 skeletal variations of triterpenoids have been described so far^17^. The most commonly encountered triterpenoids have structures comprising four or five aliphatic rings – they are therefore designated as tetracyclic and pentacyclic triterpenoids, respectively. These compounds can have a single hydroxyl functional group, though are often found with additional oxygen-containing functional groups as well. Among these diverse structures there are compounds that have antioxidant, antihistaminic, and anti-inflammatory properties^18^. So, beyond contributing to the material properties of cork, extractable triterpenoids may participate in protecting the tree from pests and have the potential to be high-value coproducts obtainable during bark processing (triterpenoids comprise about 5% of cork dry weight)^19^.

Transcriptome analyses are a widespread and powerful approach used in gene identification. Transcriptomics have helped identify *Q. suber* genes expressed in cork (phellem) whose potato orthologs biosynthesize suberin and fatty acyl compounds in tuber skin (phellem)^20–23^. Recently, RNA sequencing of cork and cork-producing cells (cork cambium or phellogen) revealed new genes potentially involved in the biosynthesis of each class of cork chemicals^24–26^, and the recent release of a draft genome for *Q. suber*^27^ provides additional resources for gene identification. However, despite the availability of these resources and the importance of triterpenoids in both cork material properties and as potential cork coproducts, no specific *Q. suber* genes for cork triterpenoid biosynthesis have yet been functionally tested. Accordingly, the objective of this work was to identify and functionally characterize genes involved in the biosynthesis of *Q. suber* cork triterpenoids. We used a chemical profiling-guided, PCR-based strategy to clone OSC candidate genes and then tested their functions via heterologous expression. We also took advantage of the transcriptomic and genomic resources available for *Q. suber* to analyze the functionally characterized genes in a genomic context, and to shed further light on the biosynthetic processes leading to cork triterpenoids. This work thus contributes to the knowledge of these biologically and commercially important compounds and provides information on enzymes that may be of interest to the pharmaceutical and biotechnology sectors.

## Results

The objective of this work was to characterize non-polar extractables, particularly triterpenoids, from cork of *Quercus suber* (2.1) and to functionally test genes controlling their production (2.2). The product specificities of the corresponding gene products were assessed in the context of homologous enzymes from other plant species (2.3), and potential redundancies in the biosynthesis of cork triterpenoids were analyzed by combining this information with a recently released *Q. suber* draft genome (2.4).

### Analysis of *Quercus suber* cork non-polar extractables

**Figure 1:**
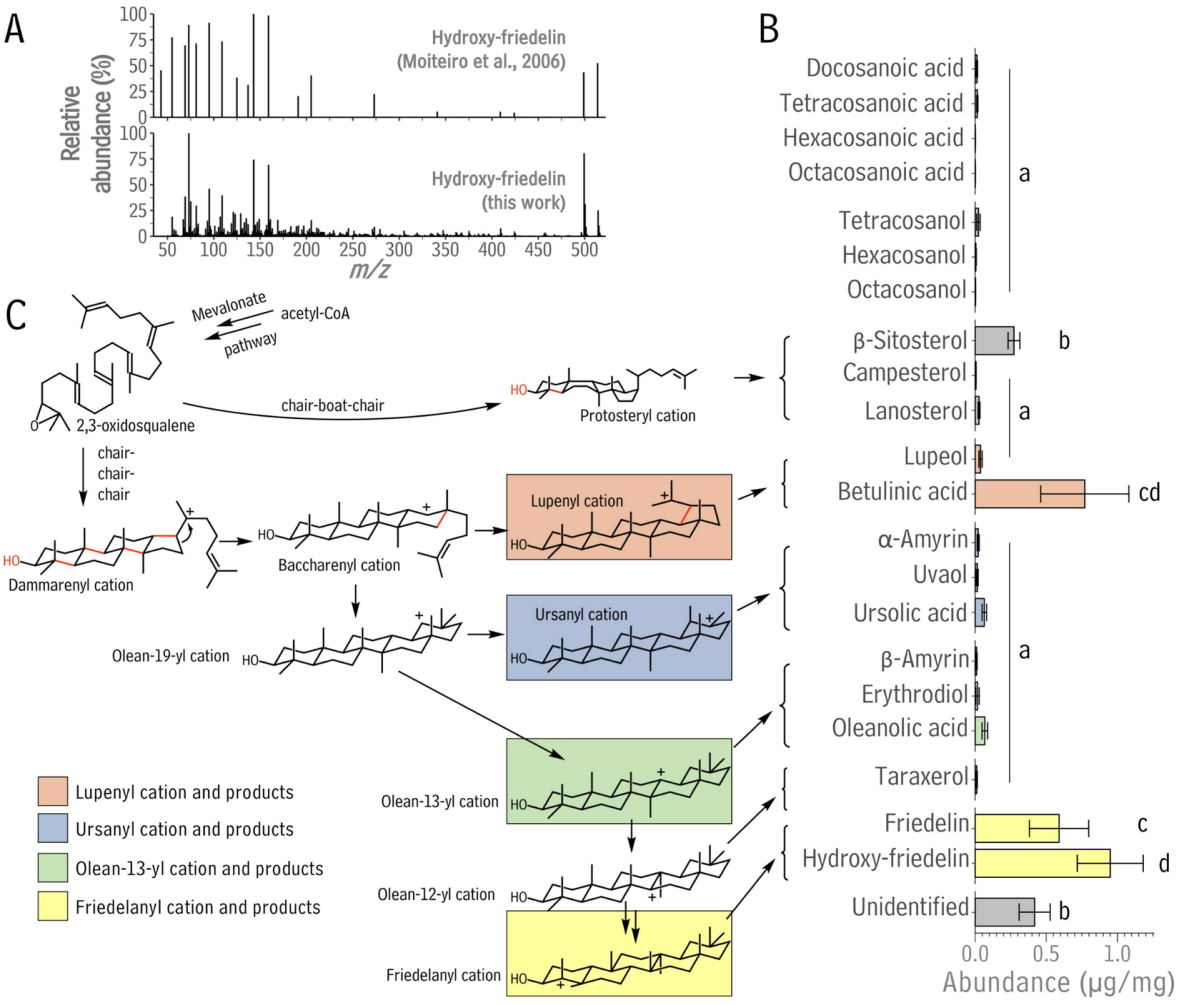
Non-polar extractable compounds from *Quercus suber* cork tissue. (**A**) Comparison of a published mass spectrum of hydroxy-friedelin (Moiteiro *et al*. 2006) and the mass spectrum of the major non-polar extractable compound in cork tissue, identified as hydroxy-friedelin. (**B)** Abundance of each wax component detected in cork in μg per mg dry tissue. Wax compounds are grouped according to their biosynthetic relationships. Bar heights and error bars represent the average and standard deviation of n = 4 biologically independent measurements. Significant differences (p < 0.01) were determined using a one-way ANOVA and subsequent Tukey Honest Significant Difference tests. (**C)** Biosynthetic routes to the triterpenoid wax compounds found in cork wax, predicted in analogy to other species (Xu *et al*., 2004). 2,3-Oxidosqualene, the precursor to triterpenoid compounds, is synthesized from six acetyl-CoA-derived isopentenyl diphosphate units supplied by the cytosolic mevalonate pathway. This compound can then be cyclized by oxidosqualene cylases to form diverse tetra- and pentacyclic structures.

To verify previous reports on non-polar extractables from cork tissue, and to assess their absolute and relative quantities, we first analyzed the contents of chloroform extracts from *Q. suber* bark (cork) in detail. The components of the extract were separated with gas chromatography (GC), then compounds were identified with mass spectrometry (MS) and quantified against an internal standard using a flame ionization detector. A total of 3.34 ± 0.94 μg material was extracted per mg dry cork tissue (Table S1). The structures of all major compounds were determined by comparing their mass spectra against those of authentic standards, except for one prominent cork constituent. The latter was identified as hydroxy-friedelin, based on its mass spectral fragmentation pattern (Supplementary Dataset 1) matching that previously reported for 23-hydroxy-friedelin^28^; Fig. 1A) and previous reports of hydroxy-friedelin in cork tissue^12^. However, the complete structure elucidation was irrelevant to the present work, and further analyses would likely be impeded by easy interconversion of isomers in the presence of an acid or base catalyst either in planta or during analysis (Fig. S6). In the cork samples, the most abundant triterpenoids were hydroxy-friedelin (0.95 ± 0.23 μg/mg) and betulinic acid (0.77 ± 0.31 μg/mg), accompanied by friedelin (0.59 ± 0.21 μg/mg), oleanolic acid (0.07 ± 0.02 μg/mg), ursolic acid (0.06 ± 0.02 μg/mg), β-sitosterol (0.27 ± 0.04 μg/mg), and trace amounts (<0.04 μg/mg each) of lupeol, β-amyrin, α-amyrin, taraxerol, erythrodiol, uvaol, lanosterol, and campesterol (Fig. 1B). Linear aliphatic compounds were also present in trace amounts, including docosanoic, tetracosanoic, hexacosanoic, and octacosanoic acids, together with tetracosanol, hexacosanol, as well as octacosanol (Fig. 1B). A small portion of the extract (12.6%; 0.42 ± 0.11 μg/mg) could not be identified.

Overall, the non-polar extractables from cork comprised exclusively very-long-chain and cyclic C_30_ aliphatic compounds also known to occur in cuticular wax mixtures covering plant epidermal tissues. The largest portion of the cork non-polar extractables (>85%) was composed of pentacyclic triterpenoids (salmon, blue, green, or yellow bars, Fig. 1B). Based on the biosynthetic pathways known to generate these compounds in other species (Fig. 1C), it was deduced that several oxidosqualene cyclases (OSCs) were likely all using 2,3-oxidosqualene as substrate to form the majority of cork triterpenoids in parallel reactions. Specifically, the chemical analyses suggested the presence of OSCs generating lupenyl, ursanyl, olean-13-yl, and friedelanyl cations (Fig. 1C), en route to respective lupeol, α-amyrin, β-amyrin, friedelin, and further downstream products.

### Identification and functional testing of putative oxidosqualene cyclases from *Quercus suber*

To identify genes encoding potential OSCs of *Q. suber*, EST libraries from cork tissue^24,29^ were screened, and specific reading frames with amino acid sequences similar to other OSCs were identified (Table S2). Using a PCR-based cloning strategy including 3′- and 5′-RACE, the full-length sequences of three OSC genes were isolated from cork cDNA (*QsOSC1:* MN428315; *QsOSC2*: MN428316: *QsOSC3*: MN428317). The amino acid sequences encoded by these genes had the following similarity percentages: QsOSC1:QsOSC2 60.7%; QsOSC1:QsOSC3 59.7%, and QsOSC2:QsOSC3 79.2%.

**Figure 2:**
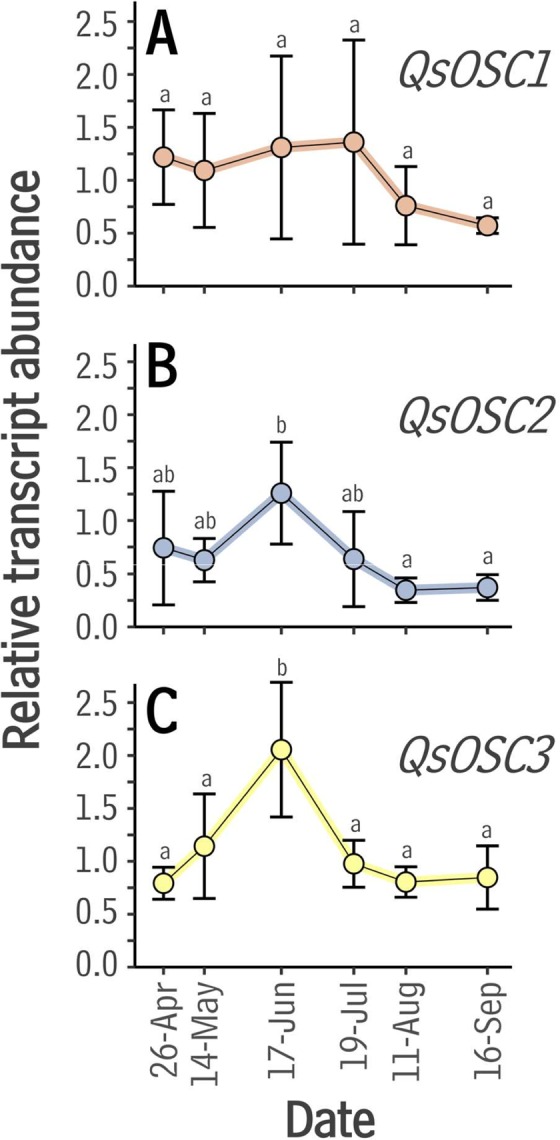
Expression of candidate OSCs in *Quercus suber* throughout the growing season as determined by RT-qPCR. The relative abundance of OSC transcripts was calculated by normalization using tubulin as the reference. Bar heights and error bars represent the mean and standard deviation of n = 4 biologically independent measurements. (**A)** *QsOSC1*, (**B)** *QsOSC2*, (**C)** *QsOSC3*. Significant differences (p < 0.05) within each time series were determined using a one-way ANOVA and subsequent Tukey Honest Significant Difference tests.

To assess the possible involvement of these OSCs in cork formation, their expression in growing cork tissue from 15- to 20-year-old trees was investigated. Reverse-transcription quantitative (Real-Time) PCR (RT-qPCR) was used to gauge the expression of these OSCs in cork tissue harvested over six consecutive months during the 2005 cork growing season in Girona, Spain, spanning the onset (April), maximum (June), and decline (September) phases of cork production^29^. *QsOSC1* was expressed at similar levels throughout the growing season (Fig. 2A), while both *QsOSC2* and *QsOSC3* were expressed most highly during June, and at lower levels thereafter (Fig. 2B,C). The temporal expression patterns of the latter two genes thus paralleled the cork growth rate, which also peaks in June^29^, indicating that these genes may play roles in cork triterpenoid accumulation.

**Figure 3:**
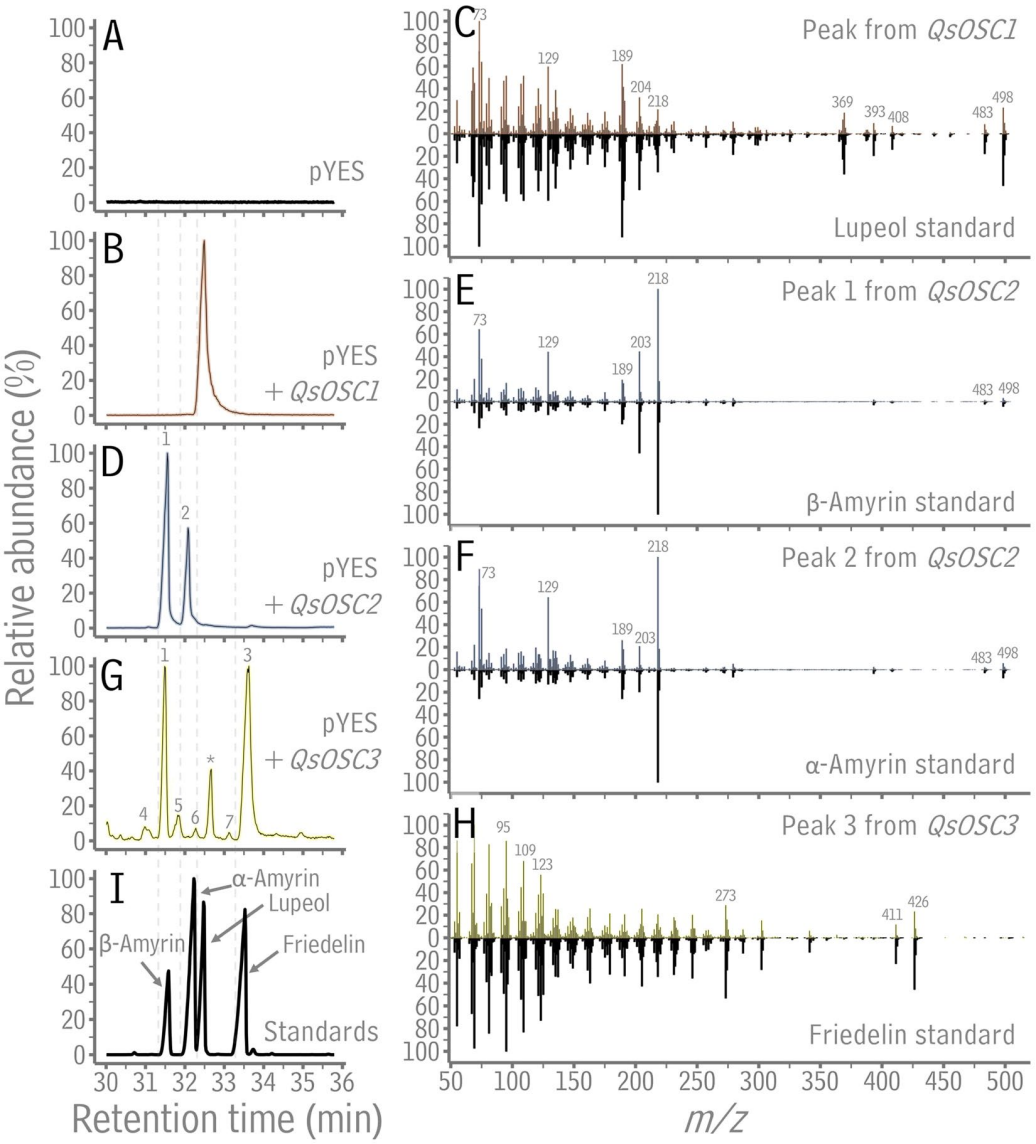
Expression of *Quercus suber* OSCs in *Saccharomyces cerevisiae*. A, B, D, G, I Total ion chromatograms of TLC-purified triterpenoid extracts of galactose-induced yeast harboring empty pYES vector (**A**), pYES::*QsOSC1* (**B**) and pYES::*QsOSC2* (**D**), total ion chromatograms of crude extracts of pYES::*QsOSC3* (**G**), and of commercial triterpenoid standards (**I**). **C**, **E**, **F**, **H** Mass spectra of the major peak in **B** and the lupeol standard (**C**), peak 1 in **D** and the β-amyrin standard (**E**), peak 2 in **D** and the α-amyrin standard (**F**), peak 3 in **G** and the friedelin standard (**H**). The mass spectra from peak 1 in **D** and peak 1 in **G** were indistinguishable. Other minor peaks (4-7) in **G** were identified as taraxerol, isomultiflorenol, lupeol, and multiflorenol, respectively, by comparison with the mass spectra of authentic standards. The peak marked with a star in **G** is an endogenous tetracyclic yeast triterpenoid (Fig. S7).

To determine the product profiles of the proteins encoded by *QsOSC1-3*, their coding sequences were inserted into a pYES-DEST52 vector and the resulting constructs were transformed into *Saccharomyces cerevisiae*. Transgene expression was induced with galactose, cells were incubated for 24 hours, then refluxed in basic conditions, and extracted with hexane. To remove endogenous yeast tetracyclic triterpenoids, the extracts were fractionated with thin-layer chromatography (TLC), and the pentacyclic triterpenoid fractions were analyzed with GC-MS. In a negative control experiment, yeast harboring empty pYES-DEST52 vector produced no pentacyclic triterpenoids (Fig. 3A). In contrast, the extract of yeast expressing *QsOSC1* contained a single pentacyclic triterpenoid with retention time and mass spectrum identical to those of lupeol (Fig. 3B,C,I). The extract from yeast harboring *QsOSC2* yielded two pentacyclic triterpenoids with GC and MS characteristics identical to those of β-amyrin and α-amyrin (Fig. 3D,E,F,I). The average ratio of β-amyrin to α-amyrin in three replicate yeast cultures was 1.9:1. The TLC plate used to fractionate the extract from yeast cells harboring *QsOSC3* bore a triterpenoid alcohol band together with a band of lesser polarity not present on the control plate, suggesting that triterpenoids with two different polarities were produced in the transgenic yeast cells. Compounds with different polarities would likely be recovered from the TLC plate with different efficiencies, which would preclude measurements of their relative abundances. Accordingly, the extract from the yeast cells harboring *QsOSC3* was analyzed in its crude form, without removing endogenous yeast tetracyclic triterpenoids by TLC fractionation. The GC-MS trace of that extract contained one tetracyclic triterpenoid peak, lanosterol, routinely detected in crude extracts from wild-type yeast cells (Fig. 3G, starred peak; Fig. S7), but also two major peaks with retention times and mass spectra identical to β-amyrin and friedelin (Fig. 3E,G,H,I). Several minor peaks, not detected in crude extracts of wild-type yeast cells, had mass spectra identifying them as the pentacyclic triterpenoids taraxerol, isomultiflorenol, lupeol, and multiflorenol by comparison with authentic standards (Fig. 3G, peaks 4-7). The average ratio of friedelin, β-amyrin, isomultiflorenol, lupeol, taraxerol, and multiflorenol across three replicate yeast cultures was 30:16:4:3:2:1. Thus, GC-MS analysis of transgenic yeast indicated that *QsOSC1* encoded an enzyme with lupeol synthase activity (*Q. suber* Lupeol synthase 1, MN428315), *QsOSC2* encoded an enzyme with both β-amyrin and α-amyrin synthase activity (*Q. suber* Multifunctional Amyrin synthase 1, MN428316), and *QsOSC3* encoded an enzyme with primarily mixed friedelin and β-amyrin synthase activity (*Q. suber* Friedelin synthase 1, MN428317). Together, the three OSC activities thus characterized can collectively form the backbones of the four major triterpenoids found in cork.

### Analysis of cloned *Quercus suber* OSC amino acid sequences

**Figure 4:**
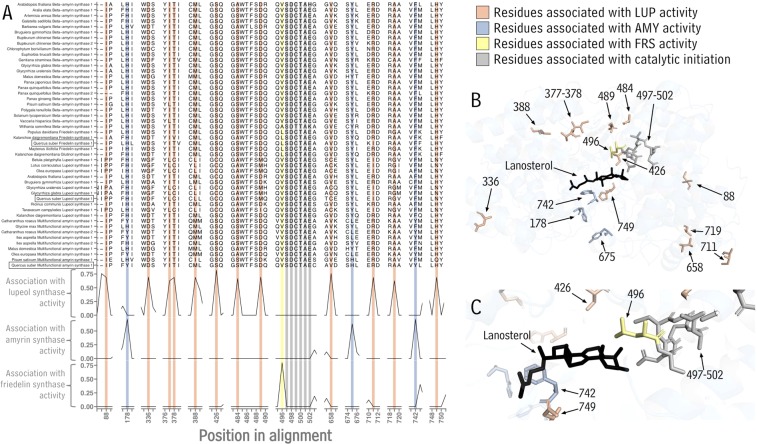
Structural analysis of *Quercus suber* oxidosqualene cyclases. (**A)** Sections of a multiple amino acid sequence alignment of OSCs from *Q. suber* and other plants. The tracks show the levels of consensus at each position in the alignment between sequences coding for specific OSC activity characterized here and sequences coding for other OSC activities. Grey highlights indicate residues associated with catalytic initiation, salmon, blue and yellow highlights indicate residues associated with lupeol, amyrin, and friedelin synthase activity, respectively. (**B)** Predicted tertiary structure of the *Q. suber* friedelin synthase *QsOSC3* with residues of interest highlighted by colors corresponding to those in (**A)**. Substrate (lanosterol) co-crystallized with the OSC template structure of human lanosterol synthase shown in black. (**C)** Active-site view of the image in **B**. (LUP = lupeol synthase; AMY = amyrin synthase; FRS = friedelin synthase).

The functional characterization of multiple OSCs (encoded by *QsOSC1*-*3*) from a single species provided an opportunity to analyze structure-function relationships in this class of enzymes. Based on our finding that the newly characterized *Q. suber* OSCs have three distinct catalytic abilities, our next objective was to identify the protein regions contributing to their different product specificities. For this, the amino acid sequences of QsOSC1-3 and triterpenoid-forming OSCs from more than 30 other plant species were aligned, and the segregation of residue identity according to OSC product specificity was assessed at each position in the alignment (Fig. S1 and Fig. 4A). The analysis revealed residues that were associated with each of the enzymatic activities of the three OSCs characterized here. Positions 88, 336, 377, 378, 388, 426, 484, 489, 658, 711, 719, and 749 in our alignment (Fig. S1) were associated with lupeol synthase activity; positions 178, 675, and 742 in our alignment were associated with the ability to synthesize a mixture of both α- and β-amyrin; and position 496 with friedelin synthase activity. The widely conserved SDCTAE motif was present in all sequences, supporting its central role in the triterpenoid cyclization mechanism, most likely through formation of the initial carbocation intermediate prompting cyclization and rearrangements^30^.

Since OSCs with friedelin synthase activity are encountered less frequently than those with, for example, amyrin synthase activity, and since friedelin synthase activity generates the majority of pentacyclic backbones for cork triterpenoid biosynthesis, the next objective was to further assess the product specificity of the friedelin synthase QsOSC3 in the context of tertiary structure. For this, the amino acid sequence of QsOSC3 was mapped onto the crystal structure of a human lanosterol synthase in complex with its substrate^31^ using Phyre2^32^ (the human lanosterol synthase is currently the only reported OSC crystal structure). The program was able to model 91% of the residues with>90% confidence, which included residues around the hydrophobic binding pocket. Based on the resulting model, it was possible to identify in the QsOSC3 structure the approximate location of the residues associated with each enzymatic function (Fig. 4B,C). The residues SDCTAE thought to initiate oxidosqualene cyclization (positions 497-502 in this alignment) lined one end of the substrate-binding pocket next to the C-1 end of the bound substrate and its epoxide group (Fig. 4B). Also near the substrate were the residues in positions 426, 496, 742, 749 of this alignment (Fig. 4C), suggesting that these particular residues determine product specificity.

### Analysis of redundancies in the biosynthesis of cork triterpenoids using a *Quercus suber* draft genome

The availability of a *Q. suber* draft genome^27^ as well as several transcriptomic datasets (Table S3) provided the ability to place the present findings into a genomic and transcriptomic context. To explore possible redundancies among OSCs forming cork triterpenoids, we used the amino acid sequences of QsOSC1-3 to query the genome with a tblastn search and filtered out hits whose longest open reading frame was less than 1,500 bp (virtually all plant OSCs characterized thus far are longer than 2,000 bp) or had gaps in the SDCTAE motif. Overall, 24 putative OSC genes were thus identified. Alignments of their nucleotide sequences with those of *QsOSC1-3* revealed that *QsOSC1* was nearly identical to XM_024037817.1, *QsOSC2* to XM_024015984.1, and *QsOSC3* to XM_024032792.1 (Fig. S2).

**Figure 5:**
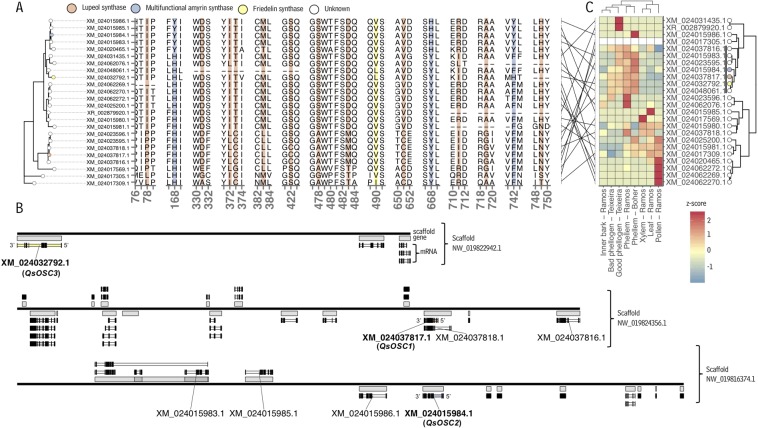
Selected amino acid motifs, scaffold locations, and expression profiles of characterized *Quercus suber* OSCs and additional predicted OSCs. (**A**) Neighbor-joining tree built using the nucleotides underlining amino acid residues shown in the adjacent alignment subsets – the positions that were associated with specific OSC activities (see Fig. 4). (**B)** Position of *Q. suber* OSCs on scaffolds of a *Q. suber* genome assembly. Black, solid lines indicate contiguous scaffolds. Features on the plus and minus strands are drawn above and below the scaffold lines, respectively. Grey rectangles immediately adjacent to scaffold lines denote boundaries of annotated genes. White (uncharacterized) or colored (characterized) rectangles above or below gene boundary markers indicate annotated mRNAs, and black rectangles superimposed on top of mRNA rectangles indicate exons. (**C)** Expression of *Q. suber* OSCs in various tissues. Rows correspond to genes and columns to Sequence Read Archive samples and both are clustered according to expression profile (z-scores calculated from transcripts per million values). Colored tree tips indicate the activity of characterized *Q. suber* OSCs (salmon = *QsOSC1*/lupeol synthase, blue = *QsOSC2*/amyrin synthase, yellow = *QsOSC3*/friedelin synthase). For detailed information on the SRA samples used see Table S3.

Using the nucleotides underlying amino acids near positions associated with OSC product specificity (Fig. 4A), a phylogenetic tree was constructed that contained all putative OSCs in the *Q. suber* genome. This revealed four distinct clades (Fig. 5A), where the genes characterized here, *QsOSC1-3*, each resided in a separate clade along with three or four other annotated OSCs. One cluster contained *QsOSC1* (XM_024037817.1) along with four putative OSCs, two of which (XM_024037816.1 and XM_024037818.1) encode residues identical to *QsOSC*1 in all the positions associated with lupeol synthase activity (Fig. 5A). The chromosomal location of the genes encoding these putative lupeol synthases indicated that XM_024037818.1 is an isoform of XM_024037817.1 (the transcript encoded by *QsOSC1*) and that the gene encoding XM_024037816.1 is located slightly upstream on the same strand and may thus be related to *QsOSC1* by tandem duplication (Fig. 5B, Fig. S4). The other two putative OSC genes in this cluster (sources of XM_024023595.1 and XM_024023596.1) also encoded identical residues in respective positions, except for a C422S substitution relative to the three genes cited above (Fig. 5A).

In the phylogenetic analysis, *QsOSC2* (XM_024015984.1) clustered with the products of three other OSC transcripts (XM_024015986.1, XM_024015985.1, and XM_024015983.1), all of which encoded identical amino acids in the positions associated with mixed α- and β-amyrin synthase activity (Fig. 5A). *QsOSC2* (XM_024015984.1) was located on the same strand and slightly upstream of the gene encoding XM_024015986.1 (Fig. 5B, Fig. S4). Furthermore, genes encoding XM_024015983.1 and XM_024015985 were located on the same scaffold, but in opposite orientation and on the opposite strand, suggesting that these two pairs of OSC genes, encoding known and putative mixed α- and β-amyrin synthases, are related by (inverted) tandem duplications.

*QsOSC3* (XM_024032792.1) clustered with products of three other annotated *Q. suber* OSC transcripts (XM_024048061.1, XM_024062076.1, and XM_024031435.1). Only the product of XM_024048061.1 had a leucine residue in the position segregating for friedelin synthase activity (490 in this alignment) (Fig. 5A), a diagnostic amino acid for friedelin synthase activity, however, this transcript was missing a substantial portion (ca. 660 bp) of the coding sequence present in each of *QsOSC1*-*3* (Fig. S3). *QsOSC3* was located on scaffold NW_019822942 and was the only OSC annotated on this scaffold.

Finally, to determine whether any of the yet uncharacterized OSCs in the *Q. suber* genome assembly may also be involved in the production of cork triterpenoids, their expression patterns were compared with those of *QsOSC1*-*3* using available RNA-Seq data. For this, data from *Q. suber* inner bark, phellogen, phellem, xylem, leaf, and pollen tissue were used^24,25,27^ (details in Table S3). *QsOSC1-3* all had very similar spatial expression patterns, with particularly high transcript specificity (zscore ≥ 0.9) in phellem, moderate expression in phellogen (0.7 ≤ zscore ≥ 0.2), and lower expression levels (zscore ≤ -0.2) in xylem, leaf, pollen, and inner bark tissues (Fig. 5C). This specific high expression of *QsOSC1*-*3* in phellem tissues suggests that their main function is in cork triterpenoid production. However, three other putative OSCs (XM_024037816.1, XM_024015983.1, XM_024023595.1) also had phellem-enhanced expression (group marked with a black line clade label, Fig. 5C), indicating that these may also have roles in cork triterpenoid production. XM_024037816.1, which results from a tandem duplication of the lupeol synthase, *QsOSC1*, encodes all the residues associated with lupeol synthase activity, indicating that it may function alongside *QsOSC1*. XM_024015983.1, the product of an inverted duplication of the multifunctional amyrin synthase, *QsOSC2*, encodes all the key residues for amyrin synthase activity, suggesting that it may operate redundantly with *QsOSC2*. XM_024023595.1 and XM_024048061.1 encode the key residues for friedelin synthase activity, though XM_024048061.1 is missing large portions of the primary sequence present in functionally validated *QsOSC3* (Fig. 5A, Fig. S3), suggesting that just XM_024023595.1 may be redundant with *QsOSC3*. Overall, these analyses strongly suggest that *QsOSC1*-*3* each function redundantly with at least one other gene in the biosynthesis of cork triterpenoids, a redundancy that further emphasizes the biological importance of triterpenoid biosynthesis in cork tissue.

## Discussion

The overall objective of this study was to use chemical profiling to guide the identification and functional characterization of genes controlling the biosynthesis of triterpenoids from *Q. suber* cork. We found that: (i) non-polar extractables from cork are composed primarily of triterpenoids, particularly those derived from the lupenyl and friedelanyl cations, (ii) three *Q. suber* oxidosqualene cyclases (*QsOSC1-3*) together catalyze the formation of lupeol, α-amyrin, β-amyrin, and friedelin, (iii) only a few OSC amino acid residues segregate with each of these OSC activities; and (iv) 20 additional OSCs in the *Q. suber* genome have sequence characteristics and expression patterns that can now be integrated to evaluate potential functional redundancy in triterpenoid biosynthesis.

We found the composition of the mixture of non-polar extractables from cork dominated (in our analyses nearly 98%) by a diverse mixture of fourteen triterpenoid compounds, with very-long-chain acyl compounds being present in very small amounts (Table S1, Fig. 1B). A previous report^12^ also described triterpenoids as major extractables from cork, alongside very-long-chain compounds present in trace amounts. This consensus indicates (i) that triterpenoid-forming pathways are much more active than pathways forming very-long-chain aliphatics dedicated to extractables and (ii) that non-polar extractables from cork are very similar to cuticular waxes from various above-ground surfaces of other plant species, thus enabling comparisons also of their biosynthesis. Similar to cork extractables, surfaces of several other plant species are known to harbor multiple triterpenoid compounds, for example, cuticular waxes from leaves of *Kalanchoe daigremontiana*^33^ and fruit of *Solanum lycopersicum*^34^. Investigations into the enzymes forming cuticular wax triterpenoids in these species revealed that multiple OSC enzyme activities underlie the production of these triterpenoid mixtures^35,36^. Based on these reports, we hypothesized the involvement of multiple OSC activities in cork terpenoid production, discovered three full-length OSC transcripts (*QsOSC1*-*3*), and determined their specific activities. QsOSC1 had lupeol synthase activity (*Q. suber* Lupeol synthase 1, MN428315), QsOSC2 had both β-amyrin and α-amyrin synthase activity (*Q. suber* Multifunctional Amyrin synthase 1, MN428316), and QsOSC3 had primarily mixed friedelin and β-amyrin synthase activity (*Q. suber* Friedelin synthase 1, MN428317). These newly characterized OSC genes, in combination with those reported previously, further underscore that gene families of substantial size are often responsible for lineage-specific metabolic processes in plants. Our findings further highlight that, while homologous genes may be identified through comparison of whole reading frames, such comparisons are not sophisticated enough to determine substrate and product specificities.

The functional characterization of multiple OSCs (*QsOSC1*-*3*) from a single species provided an opportunity for more detailed analyses of structure-function relationships in this class of enzymes. In our search for association between OSC sequences and product specificity, we identified more than ten amino acid positions in a large OSC alignment that segregated for lupeol synthase activity, three that segregated for mixed amyrin synthase activity (both α- and β-amyrin synthesis), and one that was associated with friedelin synthase activity (Fig. 4). The high number of positions associated with lupeol synthesis seems likely due to the fact that its ring system is formed by a mechanism quite distinct from those leading to the other OSC products (Fig. 1C). However, the finding that lupeol synthase sequences from diverse species cluster together in phylogenetic analyses (Fig. S5) suggests common ancestry among these enzymes, implying that some segregating residues may simply be due to this shared evolutionary history. Among the positions in our alignment that segregated for OSC product specificity, only a small number were near the active site (Fig. 4C), including residues in positions 749 and 426 (associated with lupeol synthesis), 742 (associated with multifunctional amyrin synthase activity), and 496 of our alignment (associated with friedelin synthesis). Previous sequence comparisons^37^ and point mutation studies^38,39^ demonstrated the residue in the latter position (Leu-491 in QsOSC3, position 496 in the alignment) as critical for friedelin synthesis. These results, combined with ours, underscore the power of association analyses to identify residues associated with specific catalytic activity and the extensive impact single amino acid substitutions can have on the catalytic activity of specialized metabolic enzymes.

We found that many *Q. suber OSC* genes are located together in relatively small regions of the genome (Fig. 5A, Fig. S2), including *QsOSC1* and *QsOSC2* characterized here, and that many seem to be related to one another by gene duplications. Previous studies have linked gene duplication and neofunctionalization with selective pressure in the context of specialized metabolism^40,41^, which suggests that pathways to cork triterpenoids may be under selective pressure and they play an important role in *Q. suber* biology.

**Figure 6:**
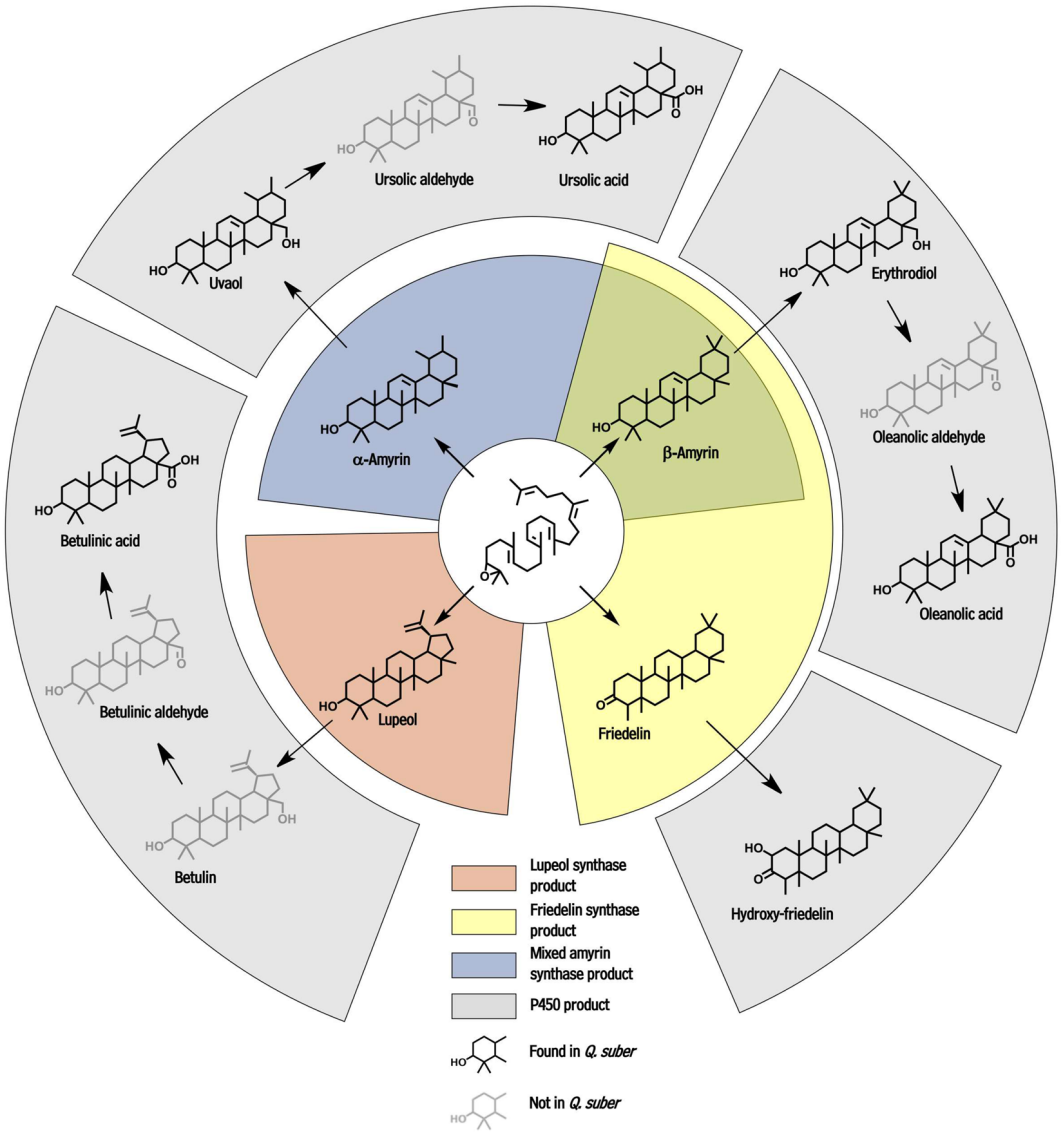
Model of triterpenoid biosynthesis in *Quercus suber* cork tissue. From squalene epoxide (in center), the *Q. suber* lupeol synthase *QsOSC1* (salmon) produces lupeol, which can then be used by P450 enzyme(s) (grey) to generate the betulinic acid detected in cork wax. The *Q. suber* amyrin synthase *QsOSC2* (blue) generates α and β-amyrin, which P450 enzyme(s) (grey) then convert into uvaol, ursolic acid, erythrodiol, and oleanolic acid. The *Q. suber* friedelin synthase *QsOSC3* (yellow) generates β-amyrin and friedelin, and the latter can then be converted into 23-hydroxy-friedelin by a P450 enzyme. Greyed structures have not been detected in cork.

Here, we characterized genes controlling the cyclization pathways that generate the major triterpenoid backbones present in non-polar extractables from cork (salmon, yellow, and blue areas, Fig. 6). However, the high relative abundance of betulinic acid and hydroxy-friedelin, as well as ursolic acid and oleanolic acid (Fig. 1B), each within their backbone class, indicate that the downstream oxidation reactions involving these backbones are also an important component of overall cork wax biosynthesis. Our data suggest that at least two P450-dependent enzymes may be involved – one that oxidizes lupeol and amyrins, and one that further hydroxylates friedelin (grey areas, Fig. 6). The OSCs identified here will be excellent seeds for future co-expression analyses aimed at identifying P450 enzymes involved in cork triterpenoid biosynthesis. Finally, we also discovered that *QsOSC2* and *QsOSC3*, a multifunctional amyrin synthase and primarily mixed friedelin and β-amyrin synthase, respectively, have particularly strong expression in June, the main month of cork growth, which indicates that transcriptional regulation may play a role in seasonal triterpenoid production in cork, and raises the important and interesting question of whether cork triterpenoids contribute to cork quality, an important agronomic trait. In summary, the genes identified here provide (i) the tools necessary for future studies to investigate both chemical and genetic questions on the role of terpenoids in cork quality, (ii) resources for triterpenoid bioengineering efforts, and (iii) seeds for future co-expression analyses aimed at identifying P450 enzymes involved in cork triterpenoid biosynthesis.

## Experimental procedures

### Isolation and GC analysis of cork wax

Phellem cells were harvested from *Quercus suber* by scratching the inside of the cork bark as reported previously^24^ and were dried to a constant weight. Four samples of phellem cells (60 mg ea.) were ground, spiked with tetracosane internal standard (10 μg), and extracted twice with chloroform (10 ml). The two extracts of each sample were combined, the solvent was evaporated, and the residue was dissolved in pyridine (20 μl) and bis-*N*,*O*-(trimethylsilyl)trifluoroacetamide (BSTFA, 20 μl). The mixture was incubated at 70 °C for 45 minutes, then the derivatization reagents were evaporated under a stream of nitrogen at 70 °C, and the residue was dissolved in chloroform (20 μl). Derivatized samples were first analyzed with GC-MS using standard 70 eV EI ionization and then with GC-FID using identical chromatographic conditions (oven temperature program: 50 °C hold for 2 min, 3 °C/min ramp to 200 °C, hold for 5 min, 3 °C/min ramp to 320, hold for 30 min), both as described previously^42^. Peaks in the GC-MS chromatograms were identified by comparing their mass spectra and retention times against those of authentic standards. Subsequently, peaks in GC-FID chromatograms were identified by comparison with GC-MS chromatograms, then peak areas in the GC-FID chromatograms were used to determine compound abundance by normalizing by the internal standard peak area and the tissue weight. Significant differences (p < 0.01) in compound abundance were determined using a one-way ANOVA and subsequent Tukey Honest Significant Difference tests implemented in R.

### RNA extraction

The cork RNA samples used for transcript relative quantification by RT-qPCR analyses were those used for previous studies^29^. Cork was harvested from trunks of 15– to 20-year-old trees at breast height in Girona, Spain (41°51′42.5″ N, 3°2′7.9″ E; UTM X = 502951; Y = 4634516.2); samples were collected during the cork growing season of 2005 (April 26, May 14, June 17, July 19, August 11 and September 16). Four trees were used as replicates at each harvest^29^. The extracted RNA was cleaned with the RNeasy MinElute Cleanup (Qiagen), and simultaneously DNA was digested on-column using DNase I (Qiagen). The RNA quantity and purity were measured using a Nanodrop spectrophotometer, and the integrity (quality) was checked by formamide-formaldehyde denaturing agarose gel electrophoresis. RNA was kept at -80 °C until use.

### Isolation of full-length cDNAs and cloning into yeast expression vectors

For *QsOSC* full-length reading frame identification and cloning, RNA extracted from the June and July cork samples was used (see above). Based on the sequences obtained from a previously described SSH library^20^ and 454-sequencing data^24^ (Table S2), we identified the ESTs of each *OSC* transcript. Those with truncated sequence (*QsOSC1* and *QsOSC3*) were completed using the 3ʹ- and/or 5’-RACE (Rapid Amplification of cDNA Ends) system from Invitrogen (ThermoFisher) following the manufacturer’s recommendations. Briefly, the cDNA pool used for the 5’-RACE was synthesized from 2.8 µg of RNA from cork treated with DNase using gene-specific primers and the SuperScript III RT (Table S2). The cDNA was then used for the 5’- and 3’-RACE procedures. The RACE PCR products encoding QsOSC1 and QsOSC3 were cloned into pCR4-TOPO vector (Invitrogen) and sequenced, the resulting full-length coding sequences were amplified from *Q. suber* cork cDNA using gene-specific primers (Table S2) and Advantage2 Polymerase (Clontech) following the manufacturer’s recommendations, and the PCR products were cloned into pCR4-TOPO vector. Since transcripts of QsOSC2 were found to be full-length in the EST data, it was cloned separately, by direct amplification with gene-specific primers (Table S2) and PrimeSTAR HS DNA Polymerase (Takara), and the PCR product was inserted into pDONR207 Gateway vector (Invitrogen). The complete full-length sequences were sequenced and deposited into GenBank with the accession numbers: MN428317 (QsFRI1), MN428315 (QsLUP1) and MN428316 (QsAMY1). Because the destination vector pYES-DEST52 for yeast protein expression is a Gateway-based vector, and to include only the coding regions, each *QsOSC1-3* open reading frame (ORF) was amplified by PCR from the original cDNA clone with gene-specific primers containing the attB recombination sites (Table S2), and the PCR products were inserted into the pDONR/Zeo donor vector to generate an entry vector following the manufacturer’s protocol. To construct the yeast expression clones for QsOSC1-3, each of the three entry clones was combined with the destination vector pYES-DEST52, and PCR was used to confirm proper cloning in the expression vectors (primers listed in Table S2) before the plasmids were used for yeast transformation.

### Functional expression of OSCs in yeast

Functional characterization was carried out in yeast mutant GIL77. Yeast transformation and insert cDNA overexpression were carried out as described previously^43^. Single clones were incubated in 20 ml synthetic complete medium without uracil, containing 20 mg/l ergosterol, 13 mg/l hemin and 5 g/l Tween 80 at 30 °C and 220 rpm for 48 hours. After induction with 2% galactose for 24 hours, cells were collected and re-suspended in the same volume of 0.1 M potassium phosphate buffer with the same supplements but lacking ergosterol and Tween 80, and incubated for one day at 30 °C. Cells from two flasks were collected into one tube, refluxed with 5 ml 20% KOH and 50% EtOH, and extracted three times with the same volume of hexane. The extracts were concentrated under a stream of nitrogen gas and then spotted onto a TLC plate (Merck, Darmstadt, Germany), which was developed with benzene:acetone (19:1, v-v). Bands were visualized by spraying with primuline and inspection under UV-light^44^. The band containing pentacyclic triterpenoids was scraped off, extracted with chloroform, and the resulting extracts were silylated with BSTFA in pyridine for 30 min at 70 °C (see above) for GC-MS analysis.

### RT-qPCR analyses

First-strand cDNA was synthesized from 1 µg of DNase-free RNA using random primers and the High-Capacity cDNA Reverse Transcription Kit with the RNase inhibitor (Applied Biosystems). For the reverse-transcription quantitative (Real-Time) PCR (RT-qPCR) analysis, gene-specific PCR primers (Table S2) were designed with Primer 3^45^. Reactions were performed in 10 µl and contained 1× LightCycler 480 SYBR Green Master I reagent (Roche), 300 nM of the respective primers, and 2.5 µl of a 75-fold dilution of the corresponding cDNA. The following standard thermal profile was used for all PCRs: 95 °C for 10 min; 40 cycles of 95 °C for 10 s and 60 °C for 1 min. Then, the melting curve analysis (95 °C for 15 s, 60 °C for 30 s and 95 °C for 15 s) confirmed the presence of a single amplicon. For each primer pair, standard curves with a 5-fold dilution series starting from 2.5-fold diluted cDNA template were obtained to determine the primer amplification efficiency. The data were visualized with the LightCycler 480 1.5 software (Roche) and exported to calculate the relative transcript abundance (RTA), where RTA = (E_target_)^ΔCt target (control–sample)^/(E_reference_)^ΔCt reference (control–sample)^, and E is the amplification efficiency for each gene^46^. Tubulin was used as a reference gene to normalize data because it was shown to be the most stable gene in cork samples^29^ and a control sample was included that contained a cDNA mixture with equal amounts of all samples. The absence of contaminant genomic DNA was verified using a negative cDNA reaction performed with no reverse transcriptase, and the absence of environmental contamination with reactions lacking template. Significant differences (p < 0.01) within each time series were determined using a one-way ANOVA and subsequent Tukey Honest Significant Difference tests implemented in R.

### Bioinformatics

Nucleotide sequences were obtained from NCBI according to the accession numbers in Table S3. The *Quercus suber* genome assembly used was ref_CorkOak1.0_top_level.gff3, downloaded from NCBI. Alignments were performed using ClustalW^47^ implemented in RStudio with the package ‘msa’^48^. *In silico* translation was carried out using the R package ‘Biostrings’. The model of the *Q. suber* friedelin synthase was generated using Phyre2^32^, which selected the crystal structure of a human lanosterol synthase^31^ as a template for the model. Phylogenetic trees were generated using the R package ‘phangorn’^49^ and visualized using the R package ‘ggtree’^50^. Transcript abundances were determined using Salmon^51^.

## Supplementary information

Supplementary Information 1.

Supplementary Information 2.
