# Supplementary material for: Oxidosqualene cyclases involved in the biosynthesis of triterpenoids in Quercus suber cork
Source: Sci Rep. 2020 May 15;10:8011. doi: 10.1038/s41598-020-64913-5 (PMC7229149; doi:10.1038/s41598-020-64913-5)
Supplement: Supplementary file 1 — Supplementary Information 1. [file 41598_2020_64913_MOESM1_ESM.docx]

**Supporting Information for**

Oxidosqualene cyclases involved in the biosynthesis of triterpenoids in *Quercus suber* cork

**Lucas Busta^1, 2,^** ^†^**, Olga Serra^3,^** ^†^**, Ok Tae Kim^4^, Marisa Molinas^3^, Irene Peré-Fossoul^3^, Mercè Figueras^3,^*, Reinhard Jetter^1, 5,^ ***

From the ^1^Department of Chemistry, University of British Columbia, 2036 Main Mall, Vancouver, BC V6T 1Z4, Canada; ^2^Center for Plant Science Innovation and Department of Biochemistry, University of Nebraska–Lincoln, Lincoln, Nebraska, 68588; ^3^Laboratori del Suro, Department of Biology, Facultat de Ciències, Universitat de Girona, Campus Montilivi sn. 17071 Girona, Spain; ^4^Department of Herbal Crop Research, National Institute of Horticultural and Herbal Science, RDA, Eumseong 369-873, South Korea; ^5^Department of Botany, University of British Columbia, 6270 University Boulevard, Vancouver, BC V6T 1Z4, Canada.

^†^ Co-first authors

**Table S1: Abundance of non-polar extractives in cork tissue.** Average (Avg) and standard deviation (St. Dev.) correspond to the values computed from four independent samples. These values are presented in µg / mg dry tissue.

|  | Abundance (µg / mg dry tissue) | | |
| --- | --- | --- | --- |
| Compound | Avg |  | St. Dev. |
| Campesterol | 0.0063 | ± | 0.0010 |
| Taraxerol | 0.011 | ± | 0.0042 |
| β-Amyrin | 0.010 | ± | 0.0035 |
| β-Sitosterol | 0.27 | ± | 0.041 |
| α-Amyrin | 0.022 | ± | 0.0069 |
| Lupeol | 0.038 | ± | 0.011 |
| Lanosterol | 0.027 | ± | 0.0070 |
| Friedelin | 0.59 | ± | 0.21 |
| 23-Hydroxy-friedelin | 0.95 | ± | 0.23 |
| Erythrodiol | 0.016 | ± | 0.012 |
| Uvaol | 0.015 | ± | 0.0062 |
| Oleanoic acid | 0.068 | ± | 0.020 |
| Betulinic acid | 0.77 | ± | 0.31 |
| Ursolic acid | 0.064 | ± | 0.016 |
| Docosanoic acid | 0.013 | ± | 0.0040 |
| Tetracosanoic acid | 0.015 | ± | 0.0040 |
| Hexacosanoic acid | 0.0015 | ± | 0.00044 |
| Octacosanoic acid | 0.0015 | ± | 0.00036 |
| Tetracosanol | 0.023 | ± | 0.012 |
| Hexacosanol | 0.0055 | ± | 0.0025 |
| Octacosanol | 0.0030 | ± | 0.00096 |
|  |  |  |  |
| Total fatty acids | 0.031 | ± | 0.0086 |
| Total fatty alcohols | 0.032 | ± | 0.014 |
| Total tetracyclics | 0.31 | ± | 0.049 |
| Total pentacyclics | 2.55 | ± | 0.77 |
|  |  |  |  |
| Total fatty acyls | 0.063 | ± | 0.021 |
| Total triterpenoids | 2.86 | ± | 0.82 |
|  |  |  |  |
| Total unidentified | 0.42 | ± | 0.11 |
|  |  |  |  |
| Total lipid | 3.34 | ± | 0.94 |

**Table S2. Primer sequences.** Template identifier and gene-specific primer sequences used to obtain *QsOSC1*-*3* cDNA full-length sequence and gene-specific primer sequences for cloning and RT-qPCR analyses.

| ***QsOSC1* (Qs Lupeol synthase 1)** | | | |
| --- | --- | --- | --- |
| Transcripts used for the oligonucleotide design | |  | EE743867 (Soler et al., 2007) |
| 5' RACE | For cDNA synthesis |  | 5’-TCCCACATCTGACAGCCAAA-3’ |
|  | For cDNA amplification |  | 5’-TGCCTCTGAATTTGGATCTTCAA-3’ |
| 3' RACE | For cDNA amplification |  | 5’-TGCATTGGAAGCGTAGAAA-3’ |
| Full-length amplification | | F | 5’-TGCCAGCCTTCTTGGTATCT-3’ |
|  |  | R | 5’-CCTTTGTTTACGCATTCACG-3’ |
| Cloning into pDONR/Zeo for yeast expression | | F | 5’-*GGGACAAGTTTGTACAAAAAAGCAGGCT*TCATGTGGAAACTGAAGATAGCC-3’ ^1^ |
|  |  | R | 5’-*GGGGACCACTTTGTACAAGAAAGCTGGGT*  CTTATGCAAATAGAACTTGCCT-3’ ^2^ |
| Reverse-transcription quantitative (Real-Time) PCR (RT-qPCR) | | F | 5’-CACAGCAGAAGGGCTAAAGG-3’ |
|  |  | R | 5’-TGGGAAACCACCATTGCTAC-3’ |
| ***QsOSC2* (Qs Multifunctional Amyrin synthase 1)** | | | |
| Transcripts used for the oligonucleotide design | |  | BAMS_BETPL_4.7, BAMS_BETPL_5.7 (Boher et al., 2018) |
| Full-length amplification | | F | 5’-*GGGACAAGTTTGTACAAAAAAGCAGGCT*GGATGTGGAAGCTGAAGATAGG-3’^1^ |
|  |  | R | 5’-*GGGGACCACTTTGTACAAGAAAGCTGGGT*GAGTTACATAGTTTTGGATGGC-3’^2^ |
| Cloning into pDONR/Zeo for yeast expression | | F | 5’-*GGGACAAGTTTGTACAAAAAAGCAGGCT*T  CATGTGGAAGCTGAAGATAGGAGA-3’ ^1^ |
|  |  | R | 5’-*GGGGACCACTTTGTACAAGAAAGCTGGGT*  CTTACATAGTTTTGGATGGCAAT-3’ ^2^ |
| Reverse-transcription quantitative (Real-Time) PCR (RT-qPCR) | | F | 5’-CCCGTTGTTTTTCCTTCCTC-3’ |
|  |  | R | 5’-AAAATCCCCATCCACCATCT-3’ |
| ***QsOSC3* (Qs Friedelin synthase 1)** | | | |
| Transcripts used for the oligonucleotide design | |  | EE743683, EE743883, EE743882 (Soler et al., 2007) |
| 5' RACE | For cDNA synthesis |  | 5’-TCCTGTTGAGGGAAATCACC-3’ |
|  | For cDNA amplification |  | 5’-CCCAGCCACCATCTTCATTT-3’ |
| Full-length amplification | | F | 5’-ATCTTGAGCCGTGCAGTTCT-3’ |
|  |  | R | 5’-TCCCATTTATTGAGGTGCATT-3’ |
| Cloning into pDONR/Zeo for yeast expression | | F | 5’-*GGGGACAAGTTTGTACAAAAAAGCAGGCT*  TCATGTGGAGGCTGAAGATAGGAG-3’ ^1^ |
|  |  | R | 5’- *GGGGACCACTTTGTACAAGAAAGCTGGGT*  CTTACATAGTTCTGGATGGCAAT-3’ ^2^ |
| Reverse-transcription quantitative (Real-Time) PCR (RT-qPCR) | | F | 5’-CCACCCAAAGCATAGGAAGA-3’ |
|  |  | R | 5’-ACCATGAACCATCAGGCATT-3’ |

^1^attB1 sequence is in Italic type.^2^attB2 sequence is in Italic type.

**Table S3: SRA data used in this work.** Details of the samples downloaded and used form the National Center for Biotechnology Information Sequence Read Archive.

| SRA Accession | Tissue | Publication |
| --- | --- | --- |
| SRR5986741 | Pollen | Ramos et al., 2018 |
| SRR5986739 | Leaf | Ramos et al., 2018 |
| SRR5986738 | Xylem | Ramos et al., 2018 |
| SRR5986740 | Inner_bark | Ramos et al., 2018 |
| SRR5986737 | Phellem | Ramos et al., 2018 |
| SRR3383083 | Phellem | Boher et al., 2018 |
| SRR1009171 | Good phellogen | Teixeira et al., 2018 |
| SRR1009172 | Bad phellogen | Teixeira et al., 2018 |

**
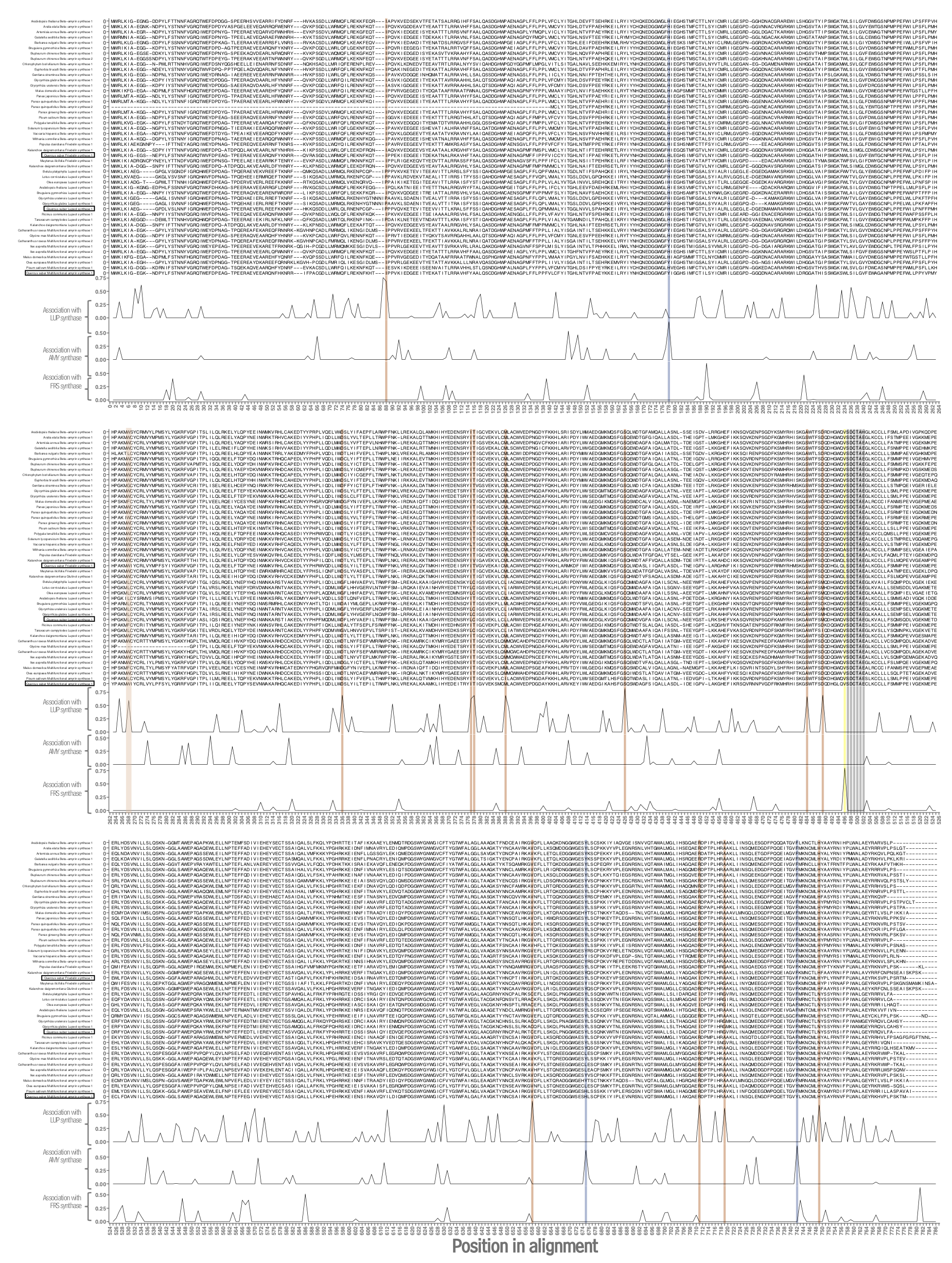
**

**Supplementary Figure 1: Multiple amino acid sequence alignment of OSCs from *Quercus suber* and other plant species.** Multiple amino acid sequence alignment of heterologously expressed *Q. suber* OSCs and other plant OSCs. The tracks show the levels of consensus at each position in the alignment between sequences coding for specific OSC activity characterized here and sequences coding for other OSC activities. Grey highlights indicate residues associated with catalytic initiation, salmon, blue and yellow highlights indicate residues associated with lupeol, amyrin, and friedelin synthase activity, respectively.

**
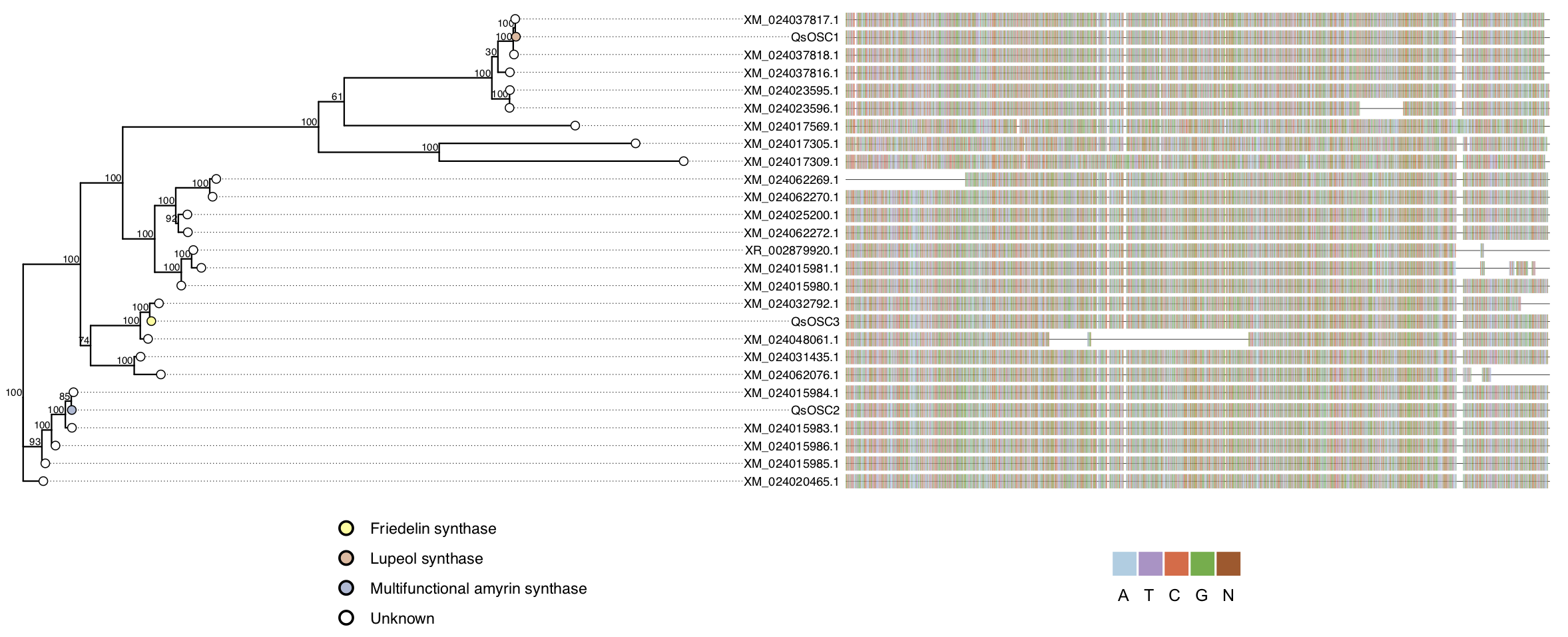
Supplementary Figure 2: Comparison of nucleotide sequences from cloned *Quercus suber* OSCs and those annotated in a *Q. suber* draft genome.** The tree indicates the relationships between the sequences at the nucleotide level. Bootstrap values indicate the number of trees out of 100 in which the corresponding node was present. Tip circle color corresponds to gene product activity indicated in the legend. The right half of the figure shows a multiple sequence alignment of the *Q. suber* sequences.

**Supplementary Figure 3: Amino acid alignment of oxidosqualene cyclases annotated in the *Quercus suber* genome.** Multiple amino acid sequence alignment of oxidosqualene cyclase homologs from *Quercus suber* that contain the SDCTAE motif associated with oxidosqualene cyclase function.

**Supplementary Figure 4: Locations of *Q. suber* OSCs on scaffolds of a draft *Q. suber* genome.** Position of *Q. suber* OSCs on scaffolds of a *Q suber* genome assembly. White rect`angles immediately adjacent to scaffold lines denote boundaries of annotated genes. White (uncharacterized) or colored (characterized) rectangles above or below gene boundary markers indicate annotated mRNAs, and black rectangles superimposed on top of mRNA rectangles indicate exons.

**Supplementary Figure 5: Maximum likelihood tree built with nucleotide sequences of functionally characterized plant OSCs.** Tip colors correspond to OSC function: green = β-amyrin synthase, blue = multifunctional amyrin synthase, orange = glutinol synthase, yellow = friedelin synthase, salmon = lupeol synthase, pink = taraxerol synthase, purple = cycloartenol synthase. Tree was rooted using the cycloartenol synthase from the moss *Physcomitrella patens*.

**Supplementary Figure 6: Potential mechanism for hydroxy-friedelin tautomerism.** Top: possible mechanism for the interconversion of 2α-OH-friedelan-3-one and 3α-OH-friedelan-2-one in the presence of an acid catalyst. Bottom: possible mechanism for the interconversion of 2α-OH-friedelan-3-one and 3α-OH-friedelan-2-one in the presence of a base catalyst.


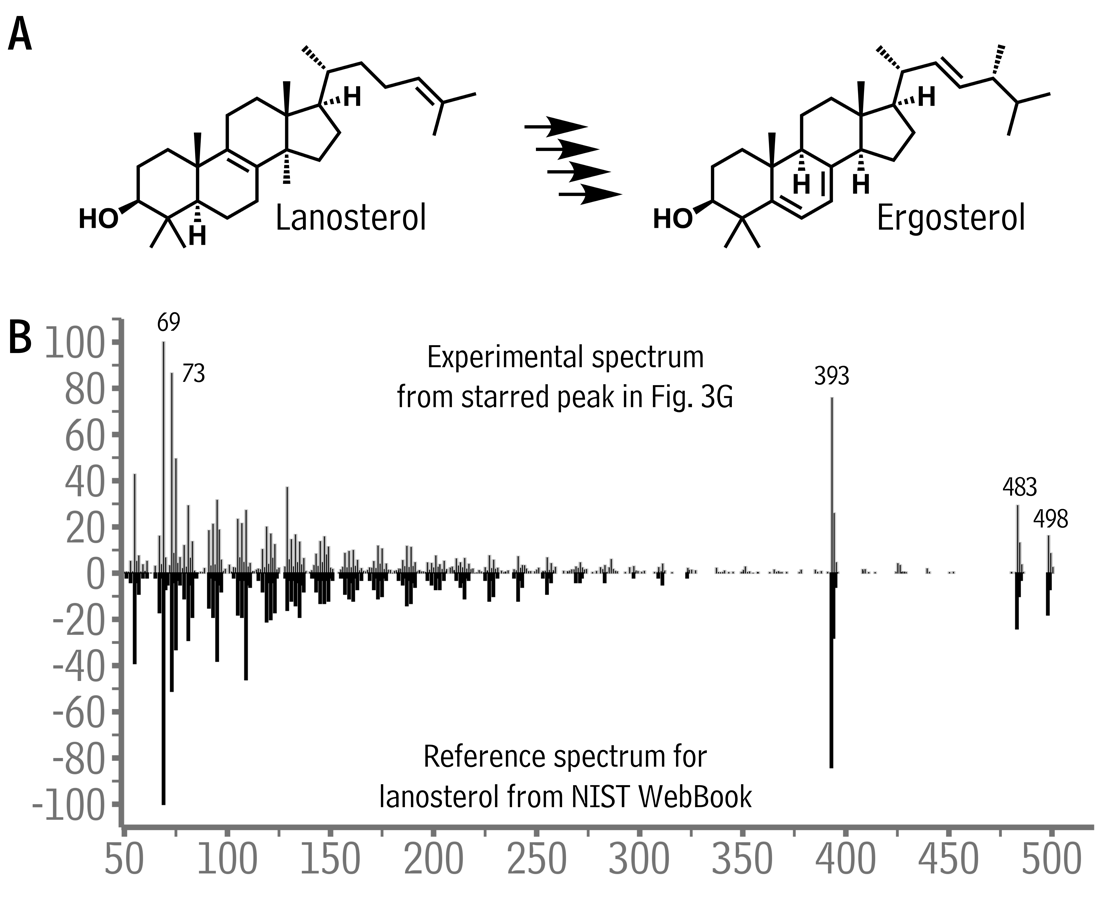
**Supplementary Figure 7: Identification of lanosterol in unpurified *Saccharomyces cerevisiae* cultures. A** Lanosterol is a ubiquitous, endogenous yeast tetracyclic triterpenoid precursor to ergosterol, a yeast sterol. **B** The mass spectrum of the starred peak in Fig. 3G (top), and the mass spectrum of lanosterol from the NIST WebBook database (bottom).
